# Voluntary exercise reduces both chemotherapy-induced neuropathic nociception and deficits in hippocampal cellular proliferation in a mouse model of paclitaxel-induced peripheral neuropathy

**DOI:** 10.1016/j.ynpai.2019.100035

**Published:** 2019-08-27

**Authors:** Richard A. Slivicki, Sonali S. Mali, Andrea G. Hohmann

**Affiliations:** a Program in Neuroscience, Indiana University, Bloomington, IN, United States; b Department of Psychological and Brain Sciences, Indiana University, Bloomington, IN, United States; c Gill Center for Biomolecular Science, Indiana University, Bloomington, IN, United States

**Keywords:** Environmental enrichment, Exercise, Neuropathic pain, Chemotherapy, Hippocampal cellular proliferation, Paclitaxel

## Abstract

Chemotherapy-induced peripheral neuropathy (CIPN) is a common dose-limiting side-effect of all major chemotherapeutic agents. Here, we explored efficacy of voluntary exercise as a nonpharmacological strategy for suppressing two distinct adverse side effects of chemotherapy treatment. We evaluated whether voluntary running would suppress both neuropathic pain and deficits in hippocampal cell proliferation in a mouse model of CIPN induced by the taxane chemotherapeutic agent paclitaxel. Mice were given free access to running wheels or were housed without running wheels during one of three different intervention phases: 1) during the onset (i.e. development phase) of paclitaxel-induced neuropathy, 2) prior to dosing with paclitaxel or its vehicle, or 3) following the establishment (i.e. maintenance phase) of paclitaxel-induced neuropathy. Paclitaxel treatment did not alter running wheel behavior relative to vehicle-treated animals in any study. Animals that engaged in voluntary running during the development phase of paclitaxel-induced neuropathy failed to display mechanical or cold hypersensitivities relative to sedentary control animals that did not have access to running wheels. A prior history of voluntary running delayed the onset of, but did not fully prevent, development of paclitaxel-induced neuropathic pain behavior. Voluntary running reduced already established mechanical and cold allodynia induced by paclitaxel. Importantly, voluntary running did not alter mechanical or cold responsivity in vehicle-treated animals, suggesting that the observed antinociceptive effect of exercise was dependent upon the presence of the pathological pain state. In the same animals evaluated for nociceptive responding, paclitaxel also reduced cellular proliferation but not cellular survival in the dentate gyrus of the hippocampus, as measured by immunohistochemistry for Ki67 and BrdU expression, respectively. Voluntary running abrogated paclitaxel-induced reductions in cellular proliferation to levels observed in vehicle-treated mice and also increased BrdU expression levels irrespective of chemotherapy treatment. Our studies support the hypothesis that voluntary exercise may be beneficial in suppressing both neuropathic pain and markers of hippocampal cellular function that are impacted by toxic challenge with chemotherapeutic agents.

## Highlights

• Paclitaxel treatment did not alter voluntary running activity.
• Voluntary running reduced mechanical and cold allodynia induced by paclitaxel.
• Voluntary running reduced paclitaxel-induced deficits in hippocampal cellular proliferation.

## 1 Introduction

Exercise has emerged as a potentially safe and cost-effective treatment for different types of chronic pain disorders (Chimenti et al., 2018, Lima et al., 2017). Recent preclinical reports suggest that exposure to treadmill or swimming exercises decreases pain responsivity in inflammatory (Terman et al., 1986, Carmody and Cooper, 1987, Kuphal et al., 2007, King-Himmelreich et al., 2017, Zheng et al., 2017), chronic muscle (Bement and Sluka, 2005) and neuropathic pain models (Stagg et al., 2011, Bobinski et al., 2015, Kim et al., 2015, Korb et al., 2010, Martins et al., 2017, Kami et al., 2016). These types of exercises allow for control over the ‘amount’ of exercise but can also introduce stress, which may alter endogenous analgesic tone, and potentially impact interpretation of results (Contarteze et al., 2008, Butler and Finn, 2009).

Mice run voluntarily when a running wheel is placed in their home cage (Lightfoot et al., 2004). Thus, voluntary wheel running represents an alternative to treadmill and swimming-based exercise that can be evaluated to assess potential therapeutic benefit of volitional exercise-induced hypoalgesia. Voluntary wheel running has produced mixed results on pain behavior depending on the timing and overall exposure time of access to running wheels and the specific pain model evaluated. Efficacy of voluntary running has been reported in antiretroviral-induced neuropathy (Ye et al., 2018), chronic muscle pain (Sabharwal et al., 2016), diabetic neuropathy (Groover et al., 2013), experimental autoimmune encephalomyelitis (Benson et al., 2015), and chronic-constriction injury (CCI) models (Grace et al., 2016), while others have reported no effect in neuropathic and inflammatory pain models (Sheahan et al., 2015).

Chemotherapy-induced peripheral neuropathy (CIPN) is a common side-effect of all major chemotherapeutic agents (Sisignano et al., 2014) and typically manifests itself in a glove and stocking distribution of allodynia. A recent report suggests that a progressive walking and resistance exercise program reduces symptoms of CIPN in people (Kleckner et al., 2018, Wonders et al., 2013). Furthermore, one report showed that treadmill exercise in mice reduced loss of intraepidermal nerve fibers induced by the paclitaxel (Park et al., 2015). However, the impact of voluntary wheel running on CIPN has never been evaluated and the possible beneficial effects of voluntary wheel running on other unwanted side effects of chemotherapy treatment remain unknown.

Chemotherapy-induced cognitive impairment (CICI) is another common problem associated with cancer chemotherapy (Ahles and Saykin, 2002). Like CIPN, CICI can persist long after the cessation of chemotherapy. In rodents, our lab and others have observed cognitive impairments following chemotherapeutic treatment that are accompanied by deficits in hippocampal cellular proliferation, suggesting a potential underlying factor driving this pathology (Panoz-Brown et al., 2017, Rendeiro et al., 2016). In addition, exercise has been reported to both suppress pathological pain (van Praag et al., 1999, Eadie et al., 2005, Smith et al., 2017) and to increase hippocampal cellular proliferation in the absence of a pathological pain state (van Praag et al., 1999, Eadie et al., 2005). However, the impact of voluntary exercise on chemotherapy-induced reductions in hippocampal cellular proliferation and survival are unknown (Smith et al., 2017).

In the present study, we used a mouse model of CIPN to explore possible therapeutic benefits of exercise on both neuropathic pain behavior and hippocampal cellular proliferation in the presence and absence of toxic challenge with paclitaxel. Paclitaxel administration results in a sustained neuropathy in approximately 30–40% of patients that can be dose-limiting, thus increasing complexity and prognosis of treatments (Cavaletti et al., 2010). Our lab (Slivicki et al., 2016, Slivicki et al., 2017, Deng et al., 2015a, Deng et al., 2015b) and others (Toma et al., 2017) have shown that paclitaxel administration results in long-lasting hypersensitivities to mechanical and cold stimulation in rodents. Therefore, we asked whether doses of paclitaxel that are sufficient to produce neuropathic nociception also induce deficits in hippocampal cellular proliferation in the same mice. We further evaluated whether voluntary exercise would produce any therapeutically beneficial impact on such deficits. In our studies, timing of exposure to running wheels was also varied to determine the optimal impact of voluntary exercise on chemotherapy-induced neuropathic nociception and chemotherapy-induced reductions in hippocampal cell proliferation. We compared three different exercise interventions, where paclitaxel or vehicle-treated mice were given access to running wheels (or no wheels) during one of three distinct temporal intervention phases either: 1) concurrent with the initiation of paclitaxel treatment; 2) under conditions in which voluntary running was initiated and terminated prior to the initiation of paclitaxel dosing; or 3) after the establishment of paclitaxel-induced neuropathic nociception.

## 2 Materials and methods

### 2.1 Materials

5-Bromo-2′-deoxyuridine (BrdU) (Sigma-Aldrich, St. Louis, MO) was dissolved in a vehicle consisting of saline and (Aquilite System; Hospira, Inc, Lake Forest, IL) containing 1 N NaOH. All animals received five once-daily injections of BrdU (100 mg/kg i.p.) beginning 26 days prior to perfusion. Paclitaxel (Tecoland, Irvine, CA) was dissolved in a vehicle consisting of cremophor (Sigma-Aldrich, St. Louis, MO), 95% ethanol (Sigma-Aldrich, St. Louis, MO) and saline in a ratio of 1:1:18, respectively, and was administered in a volume of 6.67 ml/kg.

### 2.2 Animals

Male C57BL/6J (age range 12–14 weeks at the start of experiments) mice were purchased from Jackson Laboratories (Bar Harbor, ME). Animals were maintained on a 12 h light/dark cycle with ad libitum access to food and water. All procedures were approved by the Bloomington Institutional Animal Care and Use Committee. Experimenters (RS and SM were blinded to all treatment conditions) during all facets of the study (i.e. in vivo testing, immunohistochemical experiments and quantification of histological results).

### 2.3 Voluntary exercise

Mice in the voluntary running condition were given free access to running wheels (Exercise or Ex) by placing an 11.5 cm diameter wheel in their cage without removal. The running wheel was available to the mouse 24 h/day with the exception of when animals were removed from their cages for behavioral testing. Mice in the sedentary (S, or no-running condition) were singly housed without running wheels. Running wheels were equipped with a magnetic probe (both from Mini Mitter, Bend, OR; formerly Starr Life Sciences) which counted the number of overall wheel revolutions for each mouse. Running wheel revolutions were quantified as a 24 h sum of 1-hour sample intervals.

### 2.4 Mechanical stimulation

Paw withdrawal thresholds to mechanical stimulation were evaluated as previously described (Slivicki et al., 2016, Slivicki et al., 2018). In brief, animals were habituated for at least 20 min on a wire mesh table under an inverted plastic container. Following the habituation period, an electronic von Frey anesthesiometer (IITC model Alemo 2390–5, Woodland Hills, CA) with a semi-flexible tip attached was used to stimulate the plantar surface of the hindpaw. The amount of force in grams (g) to elicit a withdrawal response (i.e. lifting of the paw) was then recorded. Measurements for each paw were performed in duplicate with at least a 7 min interval in between stimulations. These 4 values were averaged for each subject and used for data analysis.

### 2.5 Cold stimulation

Responsivity to acetone stimulation was assessed immediately after testing of mechanical paw withdrawal thresholds, as described previously (Slivicki et al., 2016, Slivicki et al., 2018). In brief, a bubble (~5–6 μL) of acetone was applied to the plantar surface of the hind paw of the animal using the blunt end of a 1 cc syringe. The animal was then observed for one min for acetone-evoked behaviors such as lifting, shaking or biting of the stimulated paw. This was performed three times per paw for each observation period, with the 6 values generated per subject at each time point subsequently averaged and used for data analysis.

### 2.6 General experimental timelines

Paclitaxel (4 mg/kg i.p.) or its vehicle was administered every other day over a period of six days (i.e. day 0, 2, 4 and 6) in all studies. In each study, day 0 denoted the beginning of paclitaxel treatment. To assess cell survival in the different treatment groups, all mice received 5 injections of BrdU (100 mg/kg i.p.) once daily on days −5 to −1 (in exercise pre-exposure or development of CIPN studies) or on days 17–21 (in exercise during maintenance of CIPN). Mice were always perfused 21 days following the last BrdU injection. In each animal, immunohistochemistry for Ki67 was used to assess the impact of the different experimental treatments on hippocampal cellular proliferation whereas immunohistochemistry for BrdU was used to assess hippocampal cell survival in the same mice.

#### 2.6.1 Study 1: exercise during development of CIPN: voluntary running during the development of paclitaxel-induced allodynia

**Fig. 1:**
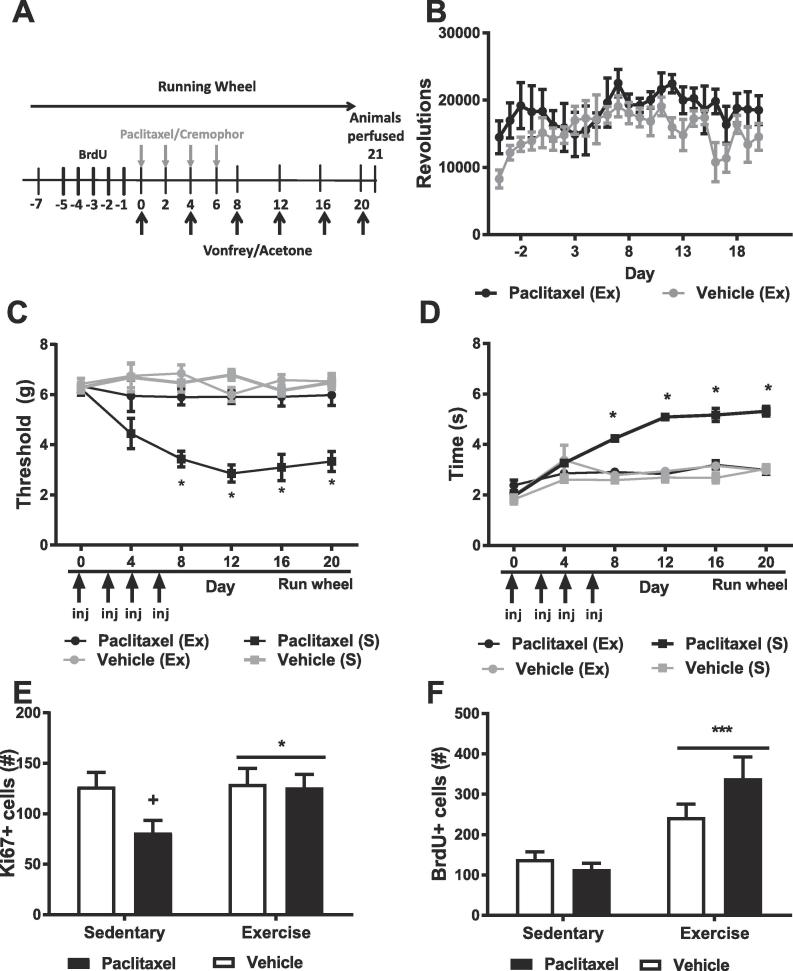
Voluntary running prevents the development of paclitaxel-induced allodynia and increases markers of cellular proliferation and survival in the dentate gyrus of the hippocampus. (A) Schematic shows timing of experimental procedures in Study 1. Voluntary running began 7 days prior to paclitaxel (4 mg/kg i.p. on day 0, 2, 4 and 6) or vehicle injections. Animals were tested for mechanical and cold hypersensitivity for a period of 20 days followed by perfusion on day 21 post paclitaxel or vehicle injection. BrdU (100 mg/kg i.p.) was administered once daily across 5 consecutive days prior to initiation of dosing with paclitaxel or its vehicle. (B) Running wheel rates did not differ in mice treated with paclitaxel or vehicle. Mice allowed to engage in voluntary exercise did not display hypersensitivities to mechanical (C) or cold (D) stimulation in contrast to sedentary mice without access to running wheels. Voluntary running did not alter responsivity to mechanical or cold stimulation in vehicle-treated animals. Paclitaxel treatment decreased the number of Ki67 (E) but not BrdU (F) labeled cells compared to vehicle treatment in sedentary mice. Voluntary running increased both Ki67 and BrdU expression levels irrespective of chemotherapy treatment condition. *p < 0.05 vs. all other groups, two-way ANOVA followed by Bonferroni post-hoc. Exercise, Ex, indicates subjects exposed to running wheels. Sedentary, S, indicates subjects housed without running wheels. (C,D) Arrows denoting injections (inj) denote timing of paclitaxel or vehicle injection. Horizontal arrow denotes timing of voluntary running. (E,F) *p < 0.05 main effect Exercise vs. Sedentary, ^+^p < 0.05 vs. vehicle, Two-Way ANOVA followed by Bonferroni post-hoc. Data are expressed as mean ± SEM. N = 5–6 per group.

We sought to evaluate how free access to running wheels before and during paclitaxel treatment would affect outcomes of distinct paclitaxel-induced toxicities. Mice were singly housed either with or without running wheels beginning 7 days (i.e. day −7) prior to receiving paclitaxel or vehicle treatment. Exposure to running wheels or no wheels continued until animals were perfused on day 21. Behavioral testing began on day 0 and continued every 4 days (see Fig. 1A). Each group in this study contained 5–6 subjects.

#### 2.6.2 Study 2: cessation of exercise prior to CIPN: impact of voluntary running prior to the onset of paclitaxel or cremophor treatment

**Fig. 2:**
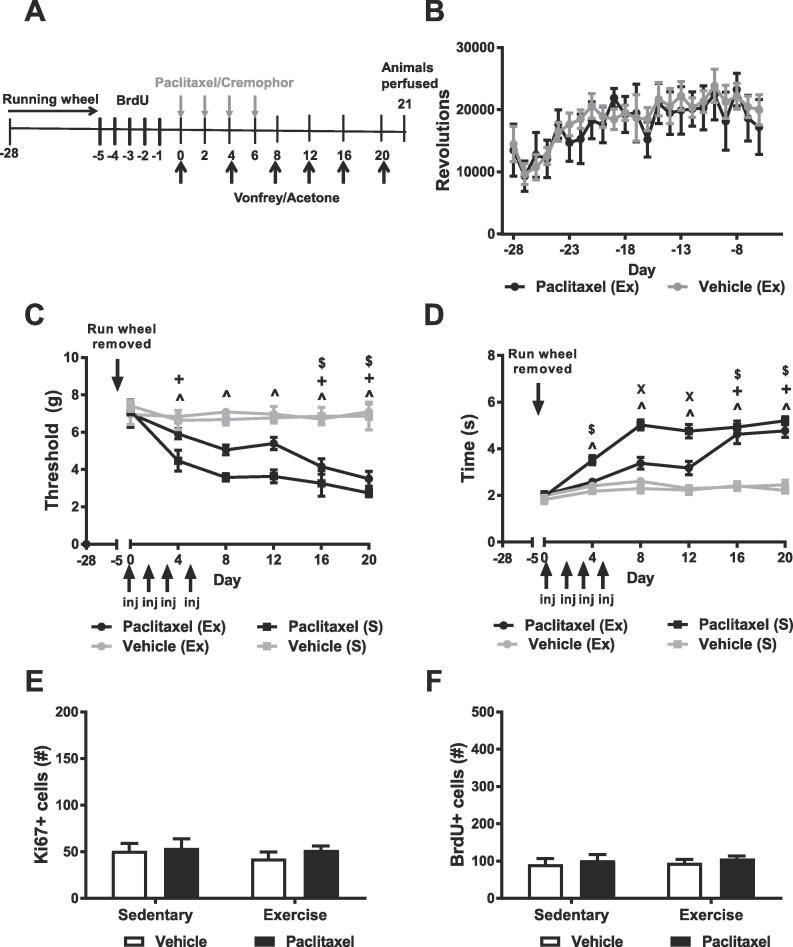
Voluntary running terminated prior to CIPN partially alleviates paclitaxel-induced allodynia. (A) Schematic shows timing of experimental procedures in Study 2. Voluntary running was initiated 28 days prior to paclitaxel or vehicle injections. Paclitaxel (4 mg/kg i.p.) or vehicle was administered on day 0, 2, 4 and 6. Animals were tested for mechanical and cold hypersensitivity for a period of 20 days followed by perfusion on day 21 post paclitaxel or vehicle injection. BrdU (100 mg/kg i.p.) was administered once daily across 5 consecutive days prior to initiation of dosing with paclitaxel or its vehicle following termination of access to running wheels or no wheels. (B) Running rates did not differ between groups prior to initiation of treatment with paclitaxel or vehicle. Prior voluntary wheel running delayed development of paclitaxel-induced (C) mechanical and (D) cold hypersensitivities. This anti-allodynic effect persisted up to 16 days following the initiation of paclitaxel dosing. The overall number of Ki67 (E) or BrdU (F) expressing cells did not differ between groups irrespective of chemotherapy or exercise condition. Voluntary running terminated (i.e. mice were given free access to running wheels and had running wheels removed 23 days later) prior to the start of paclitaxel/vehicle treatment. Data are expressed as mean ± SEM. N = 5–6 per group. ^p < 0.05 Paclitaxel Sedentary vs. Vehicle Exercise/Sedentary, ^+^p < 0.05 Paclitaxel Exercise vs. Vehicle Exercise/Sedentary, ^$^p < 0.05 paclitaxel Exercise vs. vehicle, ^X^p < 0.05 Paclitaxel Exercise vs. Paclitaxel Sedentary two-way ANOVA followed by Bonferroni post-hoc test. Exercise, Ex, indicates subjects exposed to running wheels. Sedentary, S, indicates subjects housed without running wheels. Arrows denoting injections (inj) denote timing of paclitaxel or vehicle injection. Horizontal arrow denotes timing of voluntary running.

In order to evaluate the impact of prior voluntary running wheel behavior on allodynia, hippocampal neurogenesis and cell proliferation, mice were allowed free access to running wheels for 23 days prior to paclitaxel treatment. Following the 23 day period, running wheels were removed from the home cages and mice were randomized to a treatment condition (paclitaxel or vehicle). Mechanical paw withdrawal thresholds and duration of responding to cold stimulation were evaluated every 4 days beginning the same day as paclitaxel treatment (see Fig. 2A (Lightfoot et al., 2004, Ben Abdallah et al., 2010)). Each group in this study contained 5–6 subjects.

#### 2.6.3 Study 3: exercise during the maintenance of CIPN: impact of voluntary running during the maintenance of paclitaxel-induced allodynia

**Fig. 3:**
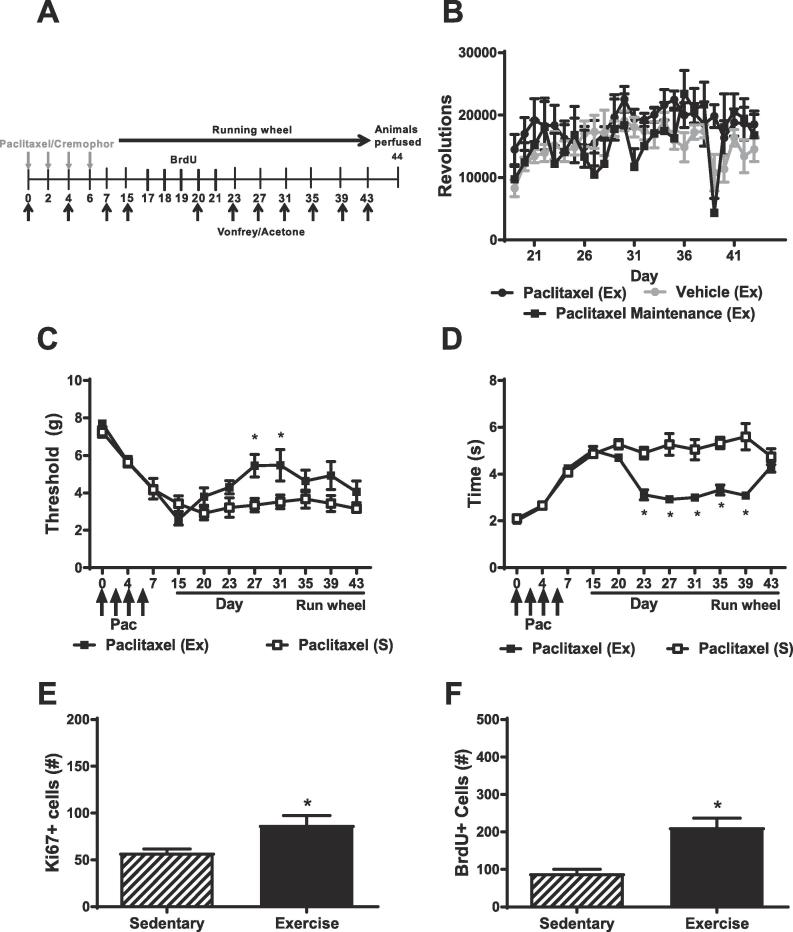
Voluntary running partially alleviates the maintenance of already established paclitaxel-induced allodynia. (A) Schematic shows timing of experimental procedures in Study 3. Voluntary running was initiated 15 days after paclitaxel (4 mg/kg i.p. on day 0, 2, 4 and 6) or vehicle injections. Mechanical and cold responsiveness was assessed prior to the start and throughout the duration of running wheel access. BrdU (100 mg/kg i.p.) was administered once daily across 5 consecutive days during the maintenance phase of paclitaxel-induced neuropathy approximately 2 days following introduction of running wheels or no wheels. Animals were perfused 44 days post paclitaxel injection. (B) Running rates did not differ in mice exposed to running wheels during the maintenance of paclitaxel-induced allodynia (paclitaxel maintenance) from those exposed concurrently with vehicle or paclitaxel treatment (paclitaxel development). Voluntary running partially attenuated paclitaxel-induced (C) mechanical and (D) cold hypersensitivities. This effect appeared to be limited to different time windows, depending on the stimulus modality assessed. Voluntary running during the maintenance phase of CIPN increased the number of Ki67 (E) and BrdU (F) expressing cells compared to paclitaxel-treated sedentary mice. Mice were exposed to running wheels for a period of 28 days or lacked access to running wheels throughout the study.*p < 0.05 vs. Paclitaxel S, two-way ANOVA followed by Bonferroni post-hoc test. Exercise, Ex, indicates subjects exposed to running wheels. Sedentary, S, indicates subjects housed without running wheels. Arrows denoting injections (inj) denote timing of paclitaxel or vehicle injection. Horizontal arrow denotes timing of voluntary running.

Paclitaxel or its vehicle was administered prior to introduction of running wheels (or no wheels) to allow paclitaxel-induced neuropathic nociception to be fully developed and maintained prior to initiation of voluntary running. Thresholds for paw withdrawal to mechanical stimulation and duration of responding to cold stimulation were assessed on day 0, 4, 7 and 15 to confirm the presence of allodynia. We previously established that on day 15 onward, paclitaxel-induced allodynia is stable and maintained for an extended observation interval (i.e. at least 2 months) (Deng et al., 2015b). Thus, on day 15 post-paclitaxel treatment, animals were housed in different cages either with or without running wheels and observed over a period of 28 days to match the previous study design (see Fig. 3A). Each group in this study contained 5–6 subjects.

### 2.7 Transcardial perfusion and brain sectioning

Animals were perfused with 4% paraformaldehyde, brains were removed and fixed overnight in 4% paraformaldehyde solution. Brains were serially sectioned (30 µM thickness) throughout the entire region of the dentate gyrus to obtain 12 sets of near adjacent sections, such that each set spanned the entire hippocampus. Sections were slide mounted, and every section in the series (i.e. every 12th section throughout the hippocampus) was quantified to assess the number of Ki67 and BrdU expressing cells, as described previously (Panoz-Brown et al., 2018).

### 2.8 Immunohistochemistry

Slides from the multiple experimental groups within a given study were processed concurrently. Slides were first washed three times with 0.1 M PBS. Following the first washes, slides underwent antigen retrieval with 10 mM sodium citrate in 0.1 M PBS. To denature DNA, slides were incubated in 2 N HCL for 30 min at 37 °C and subsequently washed in 0.1 M boric acid solution for 10 min to quench DNA denaturation. Slides were washed with PBS three times again and incubated with blocking buffer (10% normal goat serum in 0.1% Triton + PBS) for 1 h followed by primary antibody incubation. A rabbit primary Ki67 antibody (1:200, ThermoFisher Scientific, Waltham, MA) and a rat BrdU (1:200, Bio-Rad, Hercules, CA) primary antibody were incubated on slide-mounted tissue overnight. Subsequently, tissue was incubated with Alexa-Fluor anti-rabbit (1:500 dilution, 488 nm) and anti-rat (1:500 dilution, 568 nm; ThermoFisher scientific, Waltham MA) fluorescent conjugate antibodies for 2 h followed by incubation with DAPI (1:100 dilution) for 10 min. Tissue was then washed for 10 min with ddH_2_0. Tissue was mounted with Aqua-Poly/Mount (Polysciences Inc., Warrington, PA). To confirm specificity of immunofluorescence labeling, separate tissue sections were processed using methods identical to that described above with the exception that either the primary or secondary antibody, respectively, was excluded. Absence of either the primary or secondary antibody eliminated Ki67- and BrdU-like immunoreactivity (data not shown). Example photomicrographs show Ki67- and BrdU-expressing cells in the dentate gyrus of the mouse hippocampus.

### 2.9 Ki67 and BrdU quantification

The number of Ki67- and BrdU- positive cells in the granule cell layer (GCL) and subgranular zone (SGZ) were counted manually using a Leica DMLB microscope under 40x magnification by an experimenter blinded to the treatment conditions using methods described in our previously published work (Panoz-Brown et al., 2018). For quantification, 6–8 sections spanning the entire dentate gyrus of the hippocampus were quantified per animal and the total number of labeled cells per animal was calculated. The number of sections quantified did not differ between groups in any study. The number of sections quantified did not differ between groups.

### 2.10 Statistical analysis

All data analysis was performed in GraphPad Prism (GraphPad Software, La Jolla, CA), within which the following statistical tests were performed. In behavioral studies, comparisons of group differences across time were performed using two way repeated measures ANOVA. If main effects were observed, Bonferroni post hoc tests were used to evaluate the source of statistical significance. Paired t-tests were used to compare pre and post-paclitaxel baselines within subjects. The number of Ki67 and BrdU expressing cells was analyzed using 2x2 ANOVAs followed by Bonferroni post hoc tests. Bonferroni multiple comparison tests, which use the mean square result from the overall ANOVA table was also used to test a preselected subset of statistical comparisons determined by the original experimental design (Motulsky, 2014). A summary of the statistical results is present in Table 1.

**Table 1:** Summary of statistical results.

Fig. 1: Behavioral comparisons	Treatment F (df1,df2), p value	Time or Exercise Status F (df1,df2), p value	Interaction (Time × Treatment) F (df1,df2), p value

a^,^^1^Wheel Revolutions (Fig. 1B)	F_1,10_ = 3.342, p = 0.0975	F_24,240_ = 2.256, p < 0.001	F_24,240_ = 0.8518, p = 0.6678
a^,^^2^Mechanical Thresholds (Fig. 1C)	F_3,20_ = 28.25, p < 0.001	F_3,20_ = 5.193, p < 0.004	F_15,20_ = 4.379, p < 0.001
a^,^^2^Cold responsivity (Fig. 1D)	F_3,20_ = 67.17, p < 0.001	F_3,20_ = 36.02, p < 0.001	F_15,20_ = 8.432, p < 0.001

Fig. 1: Ki67 and BrdU	Paclitaxel vs. Vehicle	Exercise vs. Sedentary	Interaction

aKi67 (Fig. 1E)	F_1,18_ = 4.116, p = 0.0575	F_1,18_ = 4.503, p < 0.05	F_1,18_ = 3.113, p = 0.0946
aBrdU (Fig. 1F)	F_1,18_ = 1.338, p = 0.2625 (Interaction)	F_1,18_ = 28.83, p < 0.001	F_1,18_ = 3.911, p = 0.0635

Fig. 2: Behavioral Comparisons	Treatment	Time or Exercise Status	Interaction

a^,^^1^Wheel revolutions (Fig. 2B)	F_1,10_ = 0.079, p = 0.7841	F_22,220_ = 8.139, p < 0.001	F_22,220_ = 0.5208, p = 0.9638
a^,^^2^Mechanical Thresholds (Fig. 2C)	F_3,20_ = 47.48, p < 0.0001	F_5,20_ = 12.87, p < 0.0001	F_15,20_ = 3.891, p < 0.0001
a^,^^2^Cold Responsivity (Fig. 2D)	F_3,20_ = 64.33, p < 0.0001	F_5,20_ = 47.64, p < 0.0001	F_15,20_ = 11.41, p < 0.0001

Fig. 2: Ki67 and BrdU	Paclitaxel vs. Vehicle	Exercise vs. Sedentary	Interaction

aKi67 (Fig. 2E)	F_1,19_ = 0.6380, p = 0.4340	F_1,19_ = 0.4785, p = 0.4975	F_1,19_ = 0.1684, p = 0.6861
aBrdU (Fig. 2F)	F_1,19_ = 0.7951, p = 0.3837	F_1,19_ = 0.1068, p = 0.7474	F_1,19_ = 0.004, p = 0.9841

Fig. 3: Behavioral comparisons	Treatment	Time	Interaction

a^,^^3^Wheel revolutions (Fig. 3B)	F_2,15_ = 0.7909, p = 0.4715	F_24,360_ = 3.241, p < 0.0001	F_48,360_ = 1.278, p = 0.1120
a^,^^4^Mechanical thresholds (Fig. 3C)	F_1,10_ = 7.289, p < 0.023	F_10,10_ = 17.07, p < 0.01	F_10,10_ = 2.872, p < 0.029
a^,^^4^Cold responsivity (Fig. 3D)	F_1,10_ = 54.30, p < 0.0001	F_7,10_ = 30.09, p < 0.001	F_7,10_ = 10.91, p < 0.0001

Fig. 3: Behavioral/ Ki67 and BrdU	All groups Day 0 vs. Day 15	Paclitaxel Ex vs. Paclitaxel S	
bMechanical Thresholds (Fig. 3C)	t_11_ = 13.25, p < 0.0001 day 15 vs. day 0 mechanical thresholds		
bCold responsivity (Fig. 3D)	t_11_ = 32.32, p < 0.0001 day 15 vs. day 0 cold responsivity		
cKi67 (Fig. 3E)		t_6_ = 2.666 df = 6, p < 0.037	
cBrdU (Fig. 3F)		t_6_ = 4.567 df = 6, p < 0.003	

a Two-way ANOVA;

b Paired two-tailed *t*-test;

c Two-tailed *t*-test.

1 Paclitaxel (Exercise, Ex) vs. Vehicle (Ex).

2 Vehicle (S) vs. Vehicle (Ex) vs. Paclitaxel (Ex) vs. Paclitaxel (S).

3 Paclitaxel (Ex) vs. Paclitaxel (Ex, Fig. 1) vs. Vehicle (Ex, Fig. 1).

4 Paclitaxel (Ex) vs. Paclitaxel (S).

## 3 Results

### 3.1 Study 1: exercise during development of CIPN: voluntary running attenuates the development of paclitaxel-induced allodynia

Daily running wheel activity initiated during the development phase of neuropathy (Fig. 1A) did not differ between paclitaxel- and vehicle- treated groups, and wheel running increased over time irrespective of chemotherapy status (Fig. 1B).

Voluntary running during the development phase of neuropathy increased mechanical paw withdrawal thresholds in paclitaxel-treated mice, mechanical thresholds changed over time and the interaction between chemotherapy status and time was significant (Fig. 1C). Post-hoc comparisons revealed that sedentary paclitaxel-treated mice developed a robust hypersensitivity to mechanical stimulation relative to vehicle-treated sedentary mice (p < 0.01 for all days except 0). By contrast, mechanical thresholds did not differ in paclitaxel-treated mice that engaged in voluntary running relative to vehicle-treated animals in either exercise or sedentary conditions (p > 0.05 vs. vehicle exercise and sedentary conditions at all timepoints). Moreover, mechanical paw withdrawal thresholds were elevated in paclitaxel-treated mice that engaged in voluntary running relative to their paclitaxel-treated sedentary counterparts (p < 0.01 on days 8, 12, 16 and 20) (Fig. 1C).

Paclitaxel altered cold sensitivity, cold sensitivity differed across time and the impact of paclitaxel on cold responsivity was time dependent (Fig. 1D). Sedentary paclitaxel-treated mice developed robust hypersensitivity to acetone stimulation relative to sedentary vehicle-treated mice (p < 0.01 for all days except 0 and 4). By contrast, cold sensitivity in paclitaxel-treated mice that engaged in voluntary did not differ from vehicle-treated mice in either the exercise or sedentary conditions (p > 0.05 at all timepoints). Voluntary exercise reduced cold sensitivity in paclitaxel-treated mice relative to their paclitaxel-treated sedentary counterparts (p < 0.01 on days 8, 12, 16 and 20) (Fig. 1D).

### 3.2 Study 1: voluntary running prevents paclitaxel-induced decreases in Ki67 expression, and increases BrdU expression irrespective of treatment condition

Paclitaxel trended to reduce the number of Ki67 expressing cells observed in the dentate gyrus of the hippocampus relative to vehicle treatment (Fig. 1E). Planned comparisons revealed that paclitaxel treatment decreased the number of Ki67-expressing cells relative to vehicle treatment in sedentary mice (p < 0.05). Voluntary exercise increased the number of Ki67-expressing cells irrespective of chemotherapy treatment. The interaction between chemotherapy treatment and exercise status was not significant (Fig. 1E).

Chemotherapy treatment did not reliably alter the number of BrdU-expressing cells (Fig. 1F). Voluntary exercise increased the number of BrdU-expressing cells irrespective of chemotherapy status [and the interaction between chemotherapy treatment and exercise condition approached significance (Fig. 1F).

### 3.3 Study 2: cessation of exercise prior to CIPN: prior running wheel activity partially delays the onset of paclitaxel-induced allodynia

Running wheel activity did not differ between groups prior to the initiation of paclitaxel or vehicle treatment. Wheel running increased over time irrespective of group (Fig. 2B).

Voluntary running terminated prior to paclitaxel/vehicle treatment (Fig. 2A) increased mechanical paw withdrawal thresholds, mechanical thresholds changed across time and the interaction between chemotherapy status and time was significant (Fig. 2C). Paclitaxel-treated mice that engaged in voluntary running did not fully develop hypersensitivities until day 16 following initiation of paclitaxel dosing (p > 0.05 vs. vehicle exercise and sedentary groups on day 4, 8, 12); by day 16 this group exhibited increased mechanical sensitivity relative to vehicle-treated animals (p < 0.05 vs. vehicle-treated exercise and sedentary groups) (Fig. 2C).

Cold responsiveness differed between treatment groups, cold responsiveness changed across time and the interaction between chemotherapy status and time was significant (Fig. 2D). Mice that engaged in and terminated voluntary exercise prior to paclitaxel treatment did not fully develop cold hypersensitivities until day 16 (p > 0.05 vs. vehicle (exercise and sedentary) groups day 4, 8, 12); by day 16 this group exhibited increased cold sensitivity relative to vehicle-treated mice irrespective of exercise history (p < 0.05 vs. vehicle exercise and no exercise groups) (Fig. 2D).

### 3.4 Study 2: neither prior running wheel activity nor treatment with paclitaxel altered Ki67 or BrdU expression

The number of Ki67- (Fig. 2E) and BrdU-expressing cells (Fig. 2F) did not differ between paclitaxel- and vehicle-treatment irrespective of prior exercise history. Prior voluntary running did not alter the number of Ki67- or BrdU- expressing cells (Fig. 2E,F). The interaction between chemotherapy status and prior exercise exposure was not significant for either Ki67 or BrdU expression levels (Fig. 2E,F).

### 3.5 Study 3: voluntary exercise during the maintenance of CIPN: running wheel activity partially alleviates already established paclitaxel-induced hypersensitivities

Running rates did not differ between paclitaxel and vehicle-treated groups during the maintenance phase of paclitaxel-induced neuropathy (i.e. Fig. 3A). Running wheel activity increased over time (Fig. 3B) irrespective of treatment group.

Paclitaxel produced mechanical hypersensitivity by day 15 post-treatment (Fig. 3C). Voluntary exercise increased mechanical paw withdrawal thresholds, mechanical paw withdrawal thresholds changed over time and the impact of paclitaxel on mechanical paw withdrawal thresholds was time dependent (Fig. 3C). In paclitaxel-treated mice, voluntary running increased mechanical paw withdrawal thresholds on day 27 and 31 post-paclitaxel exposure relative to the sedentary group (p < 0.05 vs. paclitaxel sedentary group) (Fig. 3C).

Paclitaxel increased cold responsivity prior to exposure to running wheels (or no wheels), cold responsivity changed over time and the interaction was significant (Fig. 3D). Cold response times were lower in the running group compared to the sedentary group on virtually all days when animals had access to running wheels (i.e. with the exception of day 20 and 43 (p < 0.05 vs. paclitaxel sedentary) (Fig. 3D)).

### 3.6 Study 3: voluntary running during the maintenance of CIPN increases the number of Ki67 and BrdU labeled cells in paclitaxel-treated mice

**Fig. 4:**
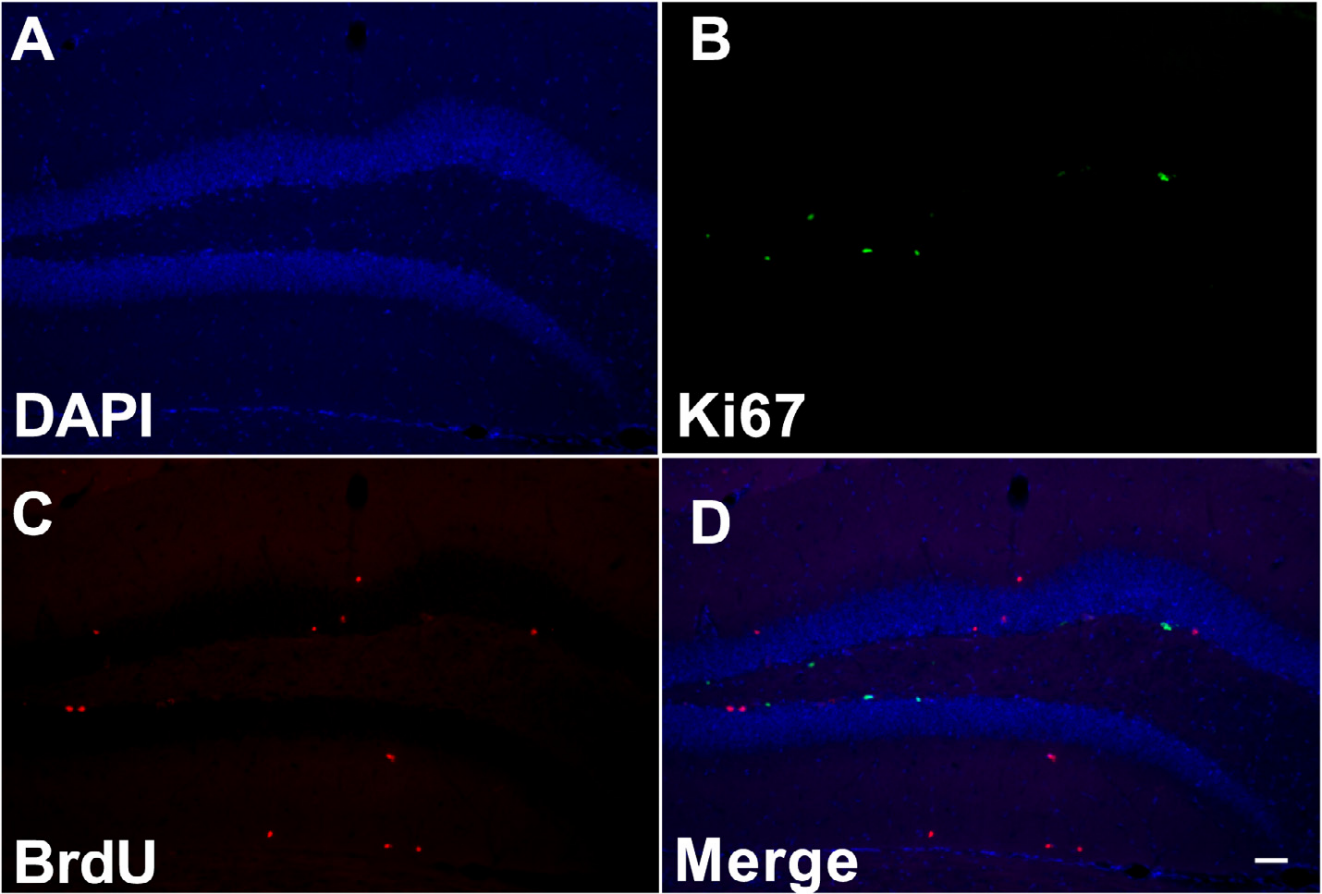
Ki67 and BrdU-like immunoreactivity in the dentate gyrus of the hippocampus. Representative photomicrographs show DAPI counterstain (blue) (A), immunoreactivity for Ki67 (green) (B), used as a marker of cellular proliferation and BrdU (red) (C), used here as a marker for cellular survival. Photomicrographs show clearly distinct expression patterns when merged (D). Photos taken at 10× magnification. Scale bar represents 200 µM. (For interpretation of the references to colour in this figure legend, the reader is referred to the web version of this article.)

Voluntary running increased the number of Ki67- (Fig. 3E) and BrdU- (Fig. 3F) expressing cells in animals exposed to running wheels during the maintenance phase of CIPN. Example photomicrographs showing Ki67- and BrdU-expressing cells in the dentate gyrus of the hippocampus are shown in Fig. 4.

## 4 Discussion

The present study is the first to evaluate the potential impact of voluntary exercise on CIPN in an animal model. We report that voluntary wheel running is therapeutically beneficial in reducing both paclitaxel-induced allodynia and paclitaxel-induced deficits in hippocampal cellular proliferation. Moreover, ongoing voluntary exercise promotes hippocampal cell survival in both paclitaxel- and vehicle-treated mice. Voluntary running throughout the development of CIPN was sufficient to fully suppress the development of paclitaxel-induced mechanical and cold allodynia relative to sedentary control animals. This conclusion is consistent with other reports showing that running wheel activity can reduce behavioral hypersensitivities in various pain models (Lima et al., 2017). Our studies support the results of a recent clinical study suggesting a therapeutic benefit of exercise for CIPN (Kleckner et al., 2018).

The impact of exercise on the development of sensory abnormalities in a model of neuropathy induced by dideoxycytidine (Ye et al., 2018) and a model of encephalomyelitis (EAE) (Benson et al., 2015) have been reported. However, the majority of reports assessing the impact on voluntary running on pain-like behavior use surgically-induced traumatic nerve injury models (i.e. where surgical recovery times preclude evaluation of impact of interventions on the development of neuropathic nociception) and/or employ unilateral injury models (i.e. where asymmetry of the inflammatory insult may impact running behavior). A unique feature of the present studies is our manipulation of the timing of running wheel access either before, during or after the onset of neuropathic nociception. Our results are the first to show that voluntary running, initiated prior to the onset of paclitaxel treatment and continuing throughout a development phase of CIPN, abolished development of paclitaxel-induced mechanical and cold allodynia throughout the observation interval (i.e. 20 days post paclitaxel). Whether voluntary exercise is capable of permanently preventing the development of paclitaxel-induced allodynia remains unknown. Our results support the hypothesis that exercise initiated prior to the onset of injury and continuing throughout its development and maintenance may be maximally efficacious in attenuating neuropathic nociception. Furthermore, because animals in our study were behaviorally tested during the light cycle when running wheel behavior is relatively low (Harri et al., 1999), the benefits of voluntary exercise are maintained even in the absence of continued active engagement in the behavior. This is in parallel to results which incorporate more transient exercise such as restricted treadmill exercise (Martins et al., 2017) and forced swim paradigms (Shen et al., 2013, Almeida et al., 2015, Kuphal et al., 2007).

Voluntary running terminated prior to the initiation of paclitaxel treatment delayed, but did not prevent, development of paclitaxel-induced allodynia irrespective of stimulus modality evaluated. By contrast, running wheel access restricted to the maintenance phase of CIPN was maximally efficacious in suppressing cold allodynia. These observations are consistent with other reports evaluating prior treadmill running in a CCI model (for 2 weeks) and voluntary running wheel activity (for 3 weeks) in a spinal cord injury model (Fu et al., 2016). A previous report demonstrated that treadmill running reduces allodynia immediately after implementation in a sciatic nerve crush model of neuropathic pain, with efficacy that is lost over time (Martins et al., 2017). Voluntary running wheel activity (for 8 weeks) also protected against hypersensitivities induced by intra-muscular acidic saline-induced chronic pain, for only a week following the insult (Sluka et al., 1985). Strikingly, exposing rats to running wheels (for 6 weeks) before induction of CCI blocked the development of allodynia for the entire observation period of 6 months (Grace et al., 2016). Differences in the duration of voluntary running, species evaluated, stimulus modality assessed and/or a difference between mechanistically distinct pathological pain states (i.e. paclitaxel and CCI) may account for differences between studies (Campbell and Meyer, 2006). Our results do not preclude the possibility that a longer duration of pre-exposure to voluntary running could permanently prevent development of paclitaxel-induced neuropathic pain.

Interestingly, voluntary running over a period of 28 days during the maintenance phase of paclitaxel-induced allodynia may be more effective at reducing cold compared to mechanical hypersensitivity. More work is necessary to determine whether this differential effect may be due, in part, to different receptor mechanisms underlying cold vs. mechanical allodynia. TRPA1 and TRPM8 are thought to be partially responsible for the gating of cold stimuli (del Camino et al., 2010), whereas responses to mechanical sensation may, in part, involve TRPA1, although other receptors such as PIEZO2 (Murthy et al., 2018), and TRPV4 contribute to the production of mechanical allodynia (Materazzi et al., 2012). In a rat model of CCI-induced neuropathic pain, the duration of running wheel access was much longer following surgery (8 weeks) than in the present study, with reductions in allodynia only beginning to be observed by week 3 of exposure and increased with each passing week of running wheel exposure (Grace et al., 2016). The therapeutic benefit of voluntary exercise may be pain model-specific, or differ based upon duration or timing of running wheel exposure given that running wheel access did not attenuate SNI-induced neuropathic pain in a prior report (Sheahan et al., 2015).

Differential therapeutic effects of voluntary exercise are likely dependent upon timing of running wheel exposure, and may reflect different neuropathological changes occurring in the periphery and CNS (Woolf and Mannion, 1999). During the development phase of paclitaxel-induced allodynia, paclitaxel accumulates in dorsal root ganglia, where it produces mitochondrial toxicity and damages primary afferents (Flatters and Bennett, 2006, Sisignano et al., 2014, Ueda, 2006, Xiao et al., 2011). The maintenance of paclitaxel-induced allodynia involves induction of proinflammatory cytokines and glial activation, processes which contribute to central sensitization (Burgos et al., 2012, Naguib et al., 2012). Macrophage entry into the DRG has also been reported following paclitaxel administration, which may contribute to the maintenance of CIPN (Jaggi and Singh, 2012). Moreover, brain resting state connectivity is also markedly altered in paclitaxel-treated rodents when allodynia is also present (Ferris et al., 2019). These mechanisms may also differ during the development and maintenance of paclitaxel-induced neuropathic nociception.

Running wheel activity has been suggested as a potential non-reflexive measure of inflammatory pain (Grace et al., 2014, Stevenson et al., 2011, Kandasamy et al., 2016, Kandasamy et al., 2017, Janelsins et al., 2010, Cobos et al., 2012). However, we found no differences in running wheel activity between neuropathic and non-neuropathic groups in any study or at any timepoint. Thus, the anti-allodynic effects of voluntary exercise observed here cannot be attributed to a change in the overall amount of wheel running between groups treated with paclitaxel or its vehicle. Our findings suggest that running wheel activity is not a valid indicator of the presence of paclitaxel-induced neuropathic nociception, consistent with other studies reporting no difference in wheel running activity in the presence or absence of other mechanistically distinct forms of neuropathic nociception (Grace et al., 2016, Sheahan et al., 2015). It is important to emphasize, however, that in our study, mice had access to running wheels for 24 h/day, and differences attributed to a pathological pain state could still be present under conditions of restricted access to running wheels.

The present paclitaxel dosing regimen reduced cell proliferation in the hippocampus. Paclitaxel treatment reduced the number of Ki67-expressing cells in the dentate gyrus of the hippocampus (i.e. in animals that were perfused at 15 weeks of age), consistent with our previous report in rats (Panoz-Brown et al., 2018) and other reports of paclitaxel-induced decreases in markers of cellular proliferation in both rats (Christie et al., 2012) and mice (Huehnchen et al., 2017). By contrast, the number of BrdU-labeled cells did not differ between paclitaxel- and vehicle-treated groups (i.e. at 20 days post BrdU administration). This observation suggests that, while paclitaxel reduced the overall number of dividing cells at this particular time point, it did not markedly alter cellular survival (i.e. at 20 days post BrdU administration). Similarly, doxorubicin and cyclophosphamide do not reduce the number of BrdU-expressing cells in rats perfused 20 days after BrdU administration (Christie et al., 2012). Using a similar BrdU injection and quantification paradigm, rats injected with cyclophosphamide or doxorubicin did not exhibit a decrease in the overall number of BrdU-expressing cells; however co-expression of BrdU with the mature neuronal marker, NeuN was reduced (Winocur et al., 2014). Our results do not preclude the possibility that the fate of the BrdU-expressing cells may have changed in response to our manipulations. Notably, ongoing voluntary running enhanced the number of Ki67- and BrdU-labeled cells irrespective of chemotherapy treatment status, verifying that running wheel exposure, on its own, enhances hippocampal cell proliferation and survival. Such effects may ameliorate paclitaxel-induced deficits in cellular proliferation.

When voluntary exercise was terminated prior to initiation of chemotherapy treatment, no differences in the numbers of Ki67- or BrdU –expressing cells were observed between groups. Thus, prior voluntary exercise may abrogate paclitaxel-induced deficits in hippocampal cellular proliferation as deficits in Ki67 expression levels were detected when voluntary exercise was initiated during the development of CIPN. Beneficial effects of voluntary exercise on BrdU expression levels were not observed in the exercise pre-exposure study (i.e. Study 2, when BrdU was administered after termination of voluntary running and before paclitaxel/vehicle treatment). Thus, paclitaxel-induced reductions in cellular proliferation, as measured by Ki67 expression levels, and cellular survival, as measured by BrdU expression levels, were not detected, suggesting that beneficial effects of prior voluntary exercise were no longer manifest at time of perfusion. Nonetheless, voluntary exercise may still blunt paclitaxel-induced deficits in cellular proliferation since such deficits were detected in sedentary animals in the development of neuropathy condition (i.e. Study 1). In another study (Lee et al., 2017), a more robust chronic paclitaxel dosing regimen (10 mg/kg every other day for 30 days) did not alter cellular proliferation in mice, as measured by Ki67 expression levels, suggesting that paclitaxel’s impact on hippocampal cellular proliferation may be dependent on many factors including age, species and treatment regimen. Beneficial effects of prior voluntary exercise on paclitaxel-induced neuropathic nociception may be more robust and pervasive than its protective effects against paclitaxel-induced deficits in hippocampal cell proliferation and survival, which, in our study, appear to require ongoing voluntary exercise

Voluntary exercise during the maintenance phase of CIPN increased both Ki67 and BrdU expression levels. These results suggest that even after the establishment of paclitaxel-induced neuropathy, voluntary running can be beneficial in enhancing both hippocampal cellular proliferation and survival. These observations have clinical relevance because deficits in hippocampal neurogenesis have been linked to chemotherapy-induced cognitive impairment (Winocur et al., 2014, Panoz-Brown et al., 2017). Moreover, prior history of voluntary running was not required for the protective effects of voluntary exercise against paclitaxel-induced losses in cellular proliferation. These observations also raise the possibility that voluntary exercise may differentially impact neuropathic pain and CICI.

Our results collectively suggest that, in line with recent clinical reports (Kleckner et al., 2018, Wonders et al., 2013), physical activity ameliorates paclitaxel-induced neuropathic pain; a view which was recently endorsed by a National Cancer Institute clinical trials planning committee (Dorsey et al., 2019). Moreover, the magnitude and pervasiveness of these beneficial effects are highly dependent on the timing of exercise and greatest under conditions in which voluntary exercise is actively maintained. We also conclude that voluntary exercise increases the number of actively proliferating cells as well as the survivability of cells within the dentate gyrus of the hippocampus even in the presence of the chemotherapeutic agent paclitaxel. Thus, regular exercise may produce mutually beneficial effects in reducing both symptoms of neuropathic pain and possible cellular correlates of cognitive impairment induced by chemotherapeutic treatment. Our studies suggest that further clinical trials assessing therapeutic benefits of voluntary exercise in patients receiving chemotherapy treatment are warranted to combat adverse toxicities associated with anti-cancer treatment.

## Declaration of Competing Interest

The authors declare that they have no known competing financial interests or personal relationships that could have appeared to influence the work reported in this paper.
